# Relationships Between Plasminogen-Binding M-Protein and Surface Enolase for Human Plasminogen Acquisition and Activation in *Streptococcus pyogenes*

**DOI:** 10.3389/fmicb.2022.905670

**Published:** 2022-05-24

**Authors:** Yetunde A. Ayinuola, Sheiny Tjia-Fleck, Bradley M. Readnour, Zhong Liang, Olawole Ayinuola, Lake N. Paul, Shaun W. Lee, Vincent A. Fischetti, Victoria A. Ploplis, Francis J. Castellino

**Affiliations:** ^1^W.M. Keck Center for Transgene Research, University of Notre Dame, Notre Dame, IN, United States; ^2^Department of Chemistry and Biochemistry, University of Notre Dame, Notre Dame, IN, United States; ^3^BioAnalysis, LLC. Philadelphia, PA, United States; ^4^Department of Biological Sciences, University of Notre Dame, Notre Dame, IN, United States; ^5^Laboratory of Bacterial Pathogenesis and Immunology, Rockefeller University, New York, NY, United States

**Keywords:** enolase, plasminogen, moonlighting proteins, scanning electron microscopy, protein mutagenesis, bacterial M-protein, *Streptococcus pyogenes*, bacterial cell surface

## Abstract

The proteolytic activity of human plasmin (hPm) is utilized by various cells to provide a surface protease that increases the potential of cells to migrate and disseminate. Skin-trophic Pattern D strains of *Streptococcus pyogenes* (GAS), e.g., GAS isolate AP53, contain a surface M-protein (PAM) that directly and strongly interacts (*K_d_* ~ 1 nM) with human host plasminogen (hPg), after which it is activated to hPm by a specific coinherited bacterial activator, streptokinase (SK2b), or by host activators. Another ubiquitous class of hPg binding proteins on GAS cells includes “moonlighting” proteins, such as the glycolytic enzyme, enolase (Sen). However, the importance of Sen in hPg acquisition, especially when PAM is present, has not been fully developed. Sen forms a complex with hPg on different surfaces, but not in solution. Isogenic AP53 cells with a targeted deletion of PAM do not bind hPg, but the surface expression of Sen is also greatly diminished upon deletion of the PAM gene, thus confounding this approach for defining the role of Sen. However, cells with point deletions in PAM that negate hPg binding, but fully express PAM and Sen, show that hPg binds weakly to Sen on GAS cells. Despite this, Sen does not stimulate hPg activation by SK2b, but does stimulate tissue-type plasminogen activator-catalyzed activation of hPg. These data demonstrate that PAM plays the dominant role as a functional hPg receptor in GAS cells that also contain surface enolase.

## Introduction

Human plasminogen (hPg) binding proteins/receptors (PgR) are cell surface proteins present on many prokaryotic and eukaryotic cells. The main functions of PgRs are to interact with hPg, stimulate its activation to the serine protease, plasmin (hPm), and protect hPm from inactivation by host inhibitors, e.g., a_2_-antiplasmin (Reinartz et al., 1993; Kramer et al., 1995; Pancholi and Fischetti, 1998). In bacteria, this accumulation of events provides cells with a potent proteolytic surface which assists in provoking an inflammatory response, as well as exacerbating their physiological and pathological dissemination and migration (Lahteenmaki et al., 1998, 2000). In *Streptococcus pyogenes*, acquisition of hPg is a key factor in its virulence (Sun et al., 2004). hPg primarily interacts with PgR *via* the lysine binding sites (LBS) of hPg kringle (K) domains. This occurs by the PgRs either providing a C-terminal lysine residue that inserts into LBS’ (Redlitz et al., 1995), or *via* through-space internal lysine isosteres that mimic a free lysine through arrangement of its amino acid side chains (Cnudde et al., 2006).

PgRs are found in many species of commensal and pathogenic Gram negative and Gram positive bacteria (Ullberg et al., 1989, 1990). The major bacterial direct binding hPg receptor is its serotype-specific M-protein, encoded by the *emm* gene. This protein coats the surface of Group A streptococci (GAS; Berge and Sjobring, 1993), a process that enhances its virulence. Approximately 250 *emm* serotypes of GAS have been identified (McMillan et al., 2013), but the M-protein (PAM) of only a subclass (Pattern D) of the GAS strains exclusively and directly interacts with hPg. The nature of the binding of hPg to PAM has been identified and involves the LBS of the K2 domain of hPg and the variable A domain of PAM (Ringdahl and Sjobring, 2000). We have provided high-resolution structures for this interaction and identified specific amino acid side chains that dictate the strong binding of these proteins to hPg (Rios-Steiner et al., 2001; Wang et al., 2010).

Other PgRs are present on cells, among, which are the glycolytic moonlighting proteins, e.g., enolase (Eno) and glyceraldehyde-3-phosphate dehydrogenase (GAPDH). While normally a cytosolic protein responsible for the penultimate step of glycolysis, Eno was found on the surface of monocytoid cells (Miles et al., 1991), neuronal membranes (Hamanoue et al., 1994), yeast cell surfaces (Edwards et al., 1999), and ubiquitously on the surfaces of streptococci (Pancholi and Fischetti, 1998; Bergmann et al., 2001; Ceremuga et al., 2014). Since Eno does not possess a signal peptide, a transmembrane domain, or other known subcellular targeting sequences, the trafficking of Eno from the cytoplasm to the cell surface is not well understood.

One major function of surface-displayed Eno is its ability to interact with the profibrinolytic precursor, hPg, *via* the C-terminal lysine residue of Eno bound to the LBS’ of K1-, K4-, and/or K5 of hPg (Miles et al., 1991; Derbise et al., 2004), or *via* an internal lysine isostere arranged from acidic and basic amino acid side chains (Redlitz et al., 1995; Bergmann et al., 2005). Here, it is believed that hPg is activated to the serine protease, hPm. This cell-bound hPm is a crucial factor in providing a mechanism for cellular migration and, in the case of pathogenic cells cellular invasion to deep tissue sites (Lahteenmaki et al., 2000; Wygrecka et al., 2009; Sawhney et al., 2015).

Pattern D strains of GAS contain both PAM and Sen and the importance of Sen in the context of GAS cells that contain PAM in terms of hPg functional binding, are not known. The clarification of the roles of PAM and Sen in this regard is the subject of this study.

## Experimental Procedures

### GAS Strains

#### Wild-Type

Group A *Streptococcus pyogenes* isolate AP53 (*emm53*; GenBank: CP106372) was obtained from Dr. G. Lindahl (Lund, SE) and used as the parent genomic DNA (gDNA) after correction of the stop codon at position 85 of the *fcR* gene to Gln, the amino acid present at that position in FcR in many strains of GAS (Liang et al., 2013). WT-SF370 (NCBI: NC002737.1) was purchased from the ATCC (ATCC700294). AP53/∆PAM and SF370/∆M1 were generated in previous studies (Liang et al., 2013).

#### AP53/PAM[8A]

To construct this mutant strain, eight residues (E^64^, K^69^, R^72^, H^73^, E^75^, K^82^, R^85^, and H^86^) in the hPg binding a1a2 repeats within the A-domain of the mature AP53/*pam* gene were replaced by alanine residues in a targeted fashion in this gene in GAS-AP53 cells (Bhattacharya et al., 2014). A PCR strategy was implemented to generate the targeting vector. Here the 3′-flank was a 601 bp PCR product spanning the PAM A-domain, from nucleotide (nt)^286^ to the PAM D-domain (nt^896^) of the AP53/*pam[8A]* cDNA, in which a silent mutation was also generated at L^58^ (ctt to ctg) to remove the *HindIII* site that existed in *pam*. This step facilitated the mapping of the final mutant strain. This DNA fragment also contained a new *PstI* site, from the R^85^ and H^86^ mutations, which further assured that PAM[R^85^A/H^86^A] existed in the mutant strain. The 5′-flank was amplified by PCR from the AP53 gDNA. The amplicon consisted of a 569 bp fragment upstream of PAM-a1a2 (nt^−266^ from the ATG of PAM) to PAM-a1a2 (nt^304^) with the same silent mutation at L^58^. The final PCR step joined the 5′-flank (nt^−266^–nt^304^) and 3′-flank (nt^286^–nt^896^), with a 19 bp overlap. This resulted in an 1,175 bp DNA fragment as the final insert that carried the *pam*[8A] mutation, along with two restriction sites, *viz., NotI* at the 5′ end and *XhoI* at the 3′ end, that are present in the genome. This fragment was inserted into a temperature-sensitive plasmid, pHY304, to obtain the targeting vector (TV). The TV was then transformed into AP53 cells using single-double crossover (SCO-DCO) as previously described (Liang et al., 2013) to generate the AP53/PAM[8A] protein in AP53 strain.

### Proteins

#### Expression and Purification of Sen and hEno

Based on the known sequence of *sen* (GenBank: AMY97107.1) from GAS strain AP53 (Bao et al., 2016), recombinant (r) Sen was generated by PCR amplification of the open reading frame encoding the *sen* gene with a 29 bp 5′-forward primer beginning at nucleotide-1 (nt)^1^ and a 31 bp 3′-reverse primer beginning at nt^1308^. Similarly, recombinant hEno was produced by amplifying the *heno* gene (NCBI: NM_001428.5) from human liver cDNA using a 28 bp 5′-forward primer beginning at nt^1^ of the reading frame and a 32 bp 3′-reverse primer beginning at nt^1308^. These primers were used in conjunction with PCR to obtain full-length *sen* and *heno*. The cDNA products were then ligated into *TopoII* plasmid and transformed into *Escherichia coli TOP10* plasmid. The *sen* and *heno* genes were then digested at their 5′ and 3′ flanks with NdeI and BamHI (the Nde1 site was placed in the primer 5′ of the ATG initiation site, and the BamH1 site was placed in the primer located 3′ of the TAA termination site). The genes were inserted into the *E. coli* expression vector, *pET-15b* (Novagen), using these same sites. The plasmids contained the ampicillin resistance gene and a N-terminal (His)_6_-tag.

Plasmids *pET15b-sen* and *pET15b-heno* were transformed into *E. coli* strain BL-21 (DE3) and grown in Luria-Bertani broth or on Luria-Bertani agar plates with ampicillin (50 μg/ml). Single bacterial colonies were grown overnight in 50 ml media and transferred to 1 L media at 37°C until an OD_600 nm_ of ~0.6 was reached (mid-log growth phase). Expression of Sen or hEno was induced with 1 mM isopropyl-ß-D-thiogalactopyranoside (IPTG) for 5 h in a shaker at 30°C. The *E. coli* cells were then collected by centrifugation, lysed, and the proteins were purified using Ni^+^-agarose affinity chromatography. Purified protein concentrations were determined by A_280nm_ using extinction coefficients of 346,600 M^−1^ cm^−1^ for Sen and 32,890 M^−1^ cm^−1^ for hEno, generated by Sednterp^1^^,^^2^ from the protein sequences based on an octameric structure for Sen and a dimeric structure for hEno. The purity of the proteins was assessed using Coomassie blue staining with SDS-PAGE gels and rabbit-anti Sen for Western analysis. The additional sequences in the non-coding regions of Sen that were needed for expression and purification are responsible for the slightly higher molecular weight of r-Sen than Sen in the cellular fractions.

#### Expression and Purification of Recombinant hPg

The cDNA encoding hPg was inserted into the pMT-Puro multiple cloning sites as previously published (Iwaki and Castellino, 2008). The resulting plasmid was transfected *into Drosophila Schneider S2* cells. Puromycin was used in the same plasmid to place positive selective pressure for production of hPg/Puro cells (Iwaki and Castellino, 2008). Those that grew under those conditions were induced with CuSO_4_ (Nilsen and Castellino, 1999). hPg in the culture medium was then purified using lysine-Sepharose affinity chromatography (Nilsen and Castellino, 1999). The hPg concentration was determined by A_280nm_, using the molar extinction coefficient 152,200 M^−1^ cm^−1^ (see footnote 1). The purity of the protein was assessed by SDS-PAGE.

#### Expression and Purification of Recombinant PAM

Plasminogen-Binding M-Protein, lacking its N-terminal signal peptide, as well as the C-terminal region containing its LPSTG CW anchor region, transmembrane binding domain, and short intracellular extension, was cloned by PCR from GAS-AP53 gDNA. A (His)_6_-tag was engineered into the reverse primer for PAM for purification by Ni+−based affinity chromatography, as described earlier (Qiu et al., 2019). This form of recombinant mature PAM thus consists of residues Asn^1^-Gln^351^, plus (His)_6_ (Bhattacharya et al., 2014).

A variant PAM that does not interact with hPg was also constructed by altering residues in the a1a2 domain such that PAM did not interact with hPg (Bhattacharya et al., 2014). This PAM variant, *viz.,*

PAM-(Asn^1^-Gln^358^/[K^58^A, K^69^A, R^72^A, H^73^A, E^74^A, K^82^A, R^85^A, H^86^A]) is designated PAM[8A]. Details of the construction, expression, purification, and properties of this mutant have been published (Bhattacharya et al., 2014).

### Rabbit Polyclonal Antibodies Against WT-Sen

Recombinant WT-Sen was used as an antigen and mixed with complete Freund’s adjuvant (CFA) or incomplete Freund’s adjuvant (IFA). Pathogen-free New Zealand White rabbits were immunized s.c. with the emulsions. Control serum was obtained from each rabbit before the antigen injection. After the first immunization with CFA, boosters were administered with IFA in 3 week intervals. Blood was taken and kept on ice for 2 h for coagulation. The serum was separated by centrifugation at 5,000 rpm for 5 min and stored at −20°C. The antibody produced was qualitatively evaluated for Sen specificity by Western blots, dot blots, and ELISA.

### Sen Enzyme Activity

The activities of Sen and hEno were monitored at room temperature in 50 mM Na phosphate/100 mM NaCl/10 mM MgCl_2_ and 3 mM 2-phosphoglycerate, pH 7.4, in a final volume of 200 μl (Pancholi and Fischetti, 1998). The conversion of 2-phosphoglycerate (2-PG) to phosphoenolpyruvate (PEP) was determined by direct assay of PEP formation at A_240nm_ (Wold and Ballou, 1957), with measurements every 5 s for a total of 3 min.

### Molecular Weight of Sen by Size Exclusion Chromatography-Multi Angle Light Scattering

This method employs Size Exclusion Chromatography (SEC) to separate components in a sample, which then pass in-line through a multi-angle light scattering apparatus (MALS), coupled to a refractive index detector, to determine the M.Wts. and concentrations, respectively, of the flow-through samples. The equipment used (Wyatt Technologies, Santa Barbara, CA, United States) consists of an Agilent 1260 Infinity II HPLC with a WTC-030S5 (7.8 mm × 300 mm, 5 μm, 300 Å) SEC column together with a Dawn HELEOS three-angle light scattering (MALS) detector coupled to an Optilab refractive index (RI) monitor. ASTRA-7 software was used for data collection and analysis. The flow path, together with the SEC column, was equilibrated with PBS, pH 7.4. Each Sen and hEno protein sample (100 μl of 1 mg/ml) was added to the column using an autosampler.

### Analytical Ultracentrifugation

Analytical Ultracentrifugation (AUC) was used to determine the sedimentation velocities (SV) and M. Wt. of Sen and hEno in native buffers. An XL-A/XL-I analytical ultracentrifuge (Beckman Coulter, United States) was used for the measurements. SV runs were performed at 30,000 rpm for Sen and 35,000 rpm for hEno using an An-60 Ti rotor and double-channel centerpiece cells at 20°C. Radial scans were recorded every 3 min for 16 h. The buffer used was 50 mM Na phosphate/0.1 M NaCl, pH 7.4, at A_280 nm_ and A_230 nm_ with three different protein dilutions. This method allows evaluation of the concentration-dependent dissociation/association of the proteins. Partial specific volumes, densities, and viscosities of the buffer were calculated from the amino acid sequences using Sedenterp.^3^ The molecular weight and sedimentation coefficients were obtained using fitting SedFit.^4^

### Binding of hPg to Immobilized Sen Using ELISA

The protein binding sites in wells of a high protein binding 96-well microtiter plates were saturated by coating with 100 μg Sen. The plate was incubated overnight at 4°C, after which the wells were washed 2x with PBS and incubated with hPg (0–1.6 mM) at 25°C for 1 h. Two additional washes with PBS were made, followed by 30 min incubations with monoclonal mouse-anti hPg conjugated to HRP (Santa Cruz Biotechnology). HRP activity was detected by a 10 min incubation with a 100 ml mixture of H_2_O_2_ and the chromogenic substrate, 3,3′,5,5′-tetramethylbenzidine (TMB; R&D Systems). The reaction was terminated by adding 2 N H_2_SO_4_ and the A_450 nm_ was determined. The saturation curve for the binding of hPg to Sen was generated using GraphPad Prism 9.0.

### Binding of hPg to Sen in Solution Using Isothermal Calorimetry

Isothermal Calorimetry (ITC) experiments were performed at 25°C in a VP-ITC 200 Microcal calorimeter (Malvern). Sen and hPg were exchanged into 100 mM phosphate pH 7.4. hPg (80 μM) was loaded in the calorimeter syringe and Sen (4 μM) was loaded in the sample cell. The differential power (DP) of the calorimeter was set to a baseline of 10 μcal/s. When the actual DP stabilized in the range of 10 ± 1 μcal/s, the titration automatically started, and data were recorded in real-time. During the titration, hPg (8 μl) was injected into SEN at a flow rate of 0.5 μl/s. In all, there were 36 injections with spacing of 180 s between each injection to ensure baseline equilibration. A reverse titration was similarly conducted with Sen (80 μM) in the syringe and hPg (4 μM) in the cell. No significant heat changes were observed in either titration.

### Binding of hPg to Immobilized Sen by Surface Plasmon Resonance

Recombinant Sen (20 μg/ml) in 10 mM NaOAc, pH 4.0, was covalently immobilized on a CM-5 chip to a target level of ~1,000 response units (RU) using the amine coupling kit (BIAcore AB). The affinity binding study was performed at 25°C in BIAcoreX100 (GE Healthcare) system with 10 mM Na^+^-Hepes/150 mM NaCl/3 mM EDTA/0.05% polysorbate 20, pH 7.4 (HBS-EP+). The chip activation, immobilization, and blocking were accomplished as previously described (Ayinuola et al., 2020). The binding experiments were done in triplicate using hPg as analyte at concentrations ranging from 9 to 2,400 nM. Each cycle employed a 10 μl/min flow rate with a 600 s association time, a 300 s dissociation time, and regeneration with 10 mM glycine-HCl, pH 1.5. The sensorgrams generated were best fit using the affinity algorithm (BIAevaluation software). For competitive binding with ε aminocaproic acid (EACA), hPg (1 mM), containing 0–50 mM EACA, were used as analytes.

### Binding of Sen to DOPG Vesicles and hPg to Sen-DOPG Vesicles Using Flow Cytometric Analysis

A frozen stock of 0.1 mM DOPG was prepared using dry solid DOPG (Millipore Sigma). DOPG was dissolved in CHCl_3_ and then dried using a rotary evaporator. The dry lipid film was resuspended in 10 mM Na-phosphate, pH 7.4, and sonicated for 5 min. The lipid vesicles were then stored at -20°C. To investigate Sen/DOPG and Sen/DOPG/hPg interactions, the frozen stock of DOPG was thawed overnight at 4°C. The next day, the PL suspension was sonicated and used for Sen experiments at 25°C. For Sen binding, 0.1 mM DOPG was first blocked in 2.5% BSA for 2 h. The PL was pelleted by centrifugation at 16,000 *g* for 10 min, after which it was resuspended in PBS, washed, and re-pelleted (Temmerman and Nickel, 2009). This was followed by a 1 h incubation in 5 μM Sen (octamer concentration), followed by a single wash step, and then incubation with polyclonal rabbit-anti Sen for 30 min. To detect the lipid-bound Sen, the PL-Sen suspension was incubated with Alexa Fluor 488-chicken-anti-rabbit IgG (Invitrogen) in the dark for 30 min. A blank experiment was similarly conducted, wherein the incubation with Sen was replaced by incubation with buffer (PBS).

For the interaction between DOPG-bound Sen and hPg, the Sen-DOPG complex was prepared as described above. After incubation with Sen, the PL was pelleted, washed, and incubated with hPg (0 or 0.8 μM) for 1 h. The pelleted PL particles were washed and then suspended in monoclonal mouse-anti hPg (ERL, South Bend, IN, United States) and incubated for 1 h. After a single wash, the PL was incubated with Alexa Fluor 488-donkey-anti-mouse IgG (Invitrogen) in the dark for 30 min. Finally, the sample was pelleted, washed, and resuspended in PBS. To exclude the interaction of hPg with PL particles, a control experiment in which the Sen incubation step was replaced with PBS, followed by incubation with hPg (0.8 μM), was performed.

Flow cytometric analysis (FCA) data were acquired at a flow rate of 10 μl/min, with 10,000 events per acquisition, using a BD FACSAria III (BD Biosciences). The lipid particles were detected by gating on fluorescence (FITC-A) with side-scatter with scales set to logarithmic amplification. Dot plots and histograms were analyzed using FCS Express Version 7 software (*De Novo* Software, Los Angeles, CA, United States). The FITC-positive gate was selected relative to the blank experiment by gating on PL particles with FITC intensity above those of the blank. The percentage of PL particles positive for FITC, and the degree of positivity (median fluorescence intensity-MFI), both of which measure the ability of Sen to interact with the PL, were recorded. The data were plotted using GraphPad Prism 9.

### Binding of hPg to PAM and Sen on GAS Cells Using FCA

WT-AP53, AP53/∆*pam*, AP53/*pam[8A]*, SF370, and SF370/∆*M1* strains were grown to early-log (OD_600nm_ = 0.3), mid-log (OD_600nm_ = 0.55), or stationary growth (OD_600nm_ = 1.0) phases. The cells were pelleted, washed once in phosphate-buffered saline (PBS), resuspended in PBS, and stored overnight at 4°C. Next, the cells were blocked in PBS/1% BSA for 30 min, and ~3 × 10^8^ cells were incubated with the proteins of interest. For the detection of PAM or Sen, the cells were incubated with rabbit-anti PAM or rabbit-anti Sen for 30 min, then pelleted, and washed 2x with PBS. Next, the cells were incubated with Alexa Fluor 488-chicken-anti-rabbit IgG (Invitrogen) in the dark for 30 min. hPg binding was assessed by incubating the cells with hPg (0, 0.2, or 2 μM) for 60 min at 25°C. The resulting cells were washed 2x with PBS and incubated for 30 min with monoclonal mouse-anti hPg IgG (ERL, South Bend, IN, United States). After washing with PBS, Alex Fluor 488-donkey-anti-mouse IgG (Invitrogen) was added and left in the dark for 30 min, after which the cells were washed 2x with PBS and fixed in PBS/1% paraformaldehyde. Acquisition of FCA data and analysis were performed as described above.

### Activation of Surface-Bound hPg

#### Plasmin Assay

The release of p-nitroaniline from S2251 was continuously monitored by A_405 nm_ for up to 120 min. The initial velocities of activation were either used directly or calculated as the slope of the linear region of a plot of A_405 nm_ vs. t^2^. The data were analyzed using GraphPad Prism Version 9.

A variety of conditions were used to activate hPg, *viz.,*

1. Solution phase activation: Replicate mixtures of hPg and Sen or PAM were incubated in microcentrifuge tubes at 25°C for 10 min, followed by the addition of S2251. The mixtures were transferred into wells of a non-protein binding 96-well microtiter plates, and hPg activation was initiated by the addition of SK2b. A 200 μl assay mixture in each well contained final concentrations of 200 nM hPg/0.8 μM Sen or 0.25 μM PAM/0.25 mM S2251/5 nM SK2b. The generation of hPm was continuously monitored by hydrolysis of S2251.
2. Immobilized hPg receptors: Sen or PAM (100 μg) was added in replicate to wells of a high protein binding 96-well plate. BSA (1%) was added to blank wells, and the plate was kept overnight at 4°C. The plate was then washed 2x with buffer to remove unbound proteins and then incubated with 400 nM hPg at 25°C for 1 h. Following this step, hPg activation was accelerated by addition of a mixture of plasminogen activator (SK1, SK2b, or tPA) and substrate, S2251, to final concentrations of 5 nM and 0.25 mM, respectively, diluting the hPg to a final concentration of 200 nM. hPm generation was continuously monitored as above. For Sen bound to DOPG, Sen (0 or 5 μM) was incubated with DOPG pre-blocked in 2.5% BSA for 1 h at 25°C. The lipid was washed, pelleted, resuspended in PBS, and then added to wells of a non-protein binding 96-well microtiter plate and incubated with 400 nM hPg for 1 h at 25°C. Activation of hPg was initiated as above.
3. Whole cell assays: Mid-log phase GAS cells were blocked in PBS/1% BSA for 30 min. GAS cells (~2 × 10^8^) were added to wells of a non-protein binding 96-well microtiter plate and incubated with 400 nM hPg 25°C for 15 min. Activation of hPg was initiated as in the Immobilized hPg receptors above.

#### Hyaluronic acid (Hya) Capsule Assays

These assays were performed as described previously (Liang et al., 2013) with operational modifications. Ten mL of washed mid-log GAS cells were pelleted and resuspended in 500 μl of H_2_O. Next, 60 µL of each cell suspension was set aside on ice for serial dilutions to 10^−7^, plated in duplicate on THY-agar plates, and incubated overnight for CFU counting. One mL of CH_3_Cl was added to the remaining 440 µL of cell suspensions. The resulting samples were shaken vigorously for 5 min at room temperature, and then vortexed for 30 sec to ensure full capsule release. The cell suspensions were then centrifuged in a microcentrifuge at max speed for 10 min. The aqueous upper phase containing the Hya capsule was then collected.

Experimental samples were prepared in triplicate in 96-well microtiter plates with 75 µL of H_2_O and 75 µL of capsule sample. These wells were then titrated 1:2, 1:4, and 1:8. A set of hyaluronic acid standards were also prepared in triplicate in the plate from 0–250 µg/mL. Next, 100 µL of stains-all solution (1-ethyl-2-[3-(1-ethylnaphtho-[1,2-d]thiazolin-2-ylidene)-2-methylpropenyl]naptho-[1,2-d]thiazolium bromide (Sigma-Aldrich)) containing 4 mg stains-all/12 µL glacial acetic acid/20 mL 50% formamide in H_2_O was added to each well and the A_640_ nm was measured. The absorbances of the Hya standards were used to generate a standard curve which was used to calculate the hyaluronic acid content of the bacteria sample wells in Hya/fg/CFU of GAS cells. Three independent runs of the experiment were performed, and the data collected as mean ± SEM using PRISM 9.

### qT-PCR of AP53-RNA

AP53, AP53/ΔPAM, and AP53-PAM[8A] GAS strains, were grown aerobically in Todd-Hewitt (TH) broth supplemented with 0.2% yeast extract (THY medium) at 37°C. Mid-log phase cells (OD_600_ of ~0.55) were collected and AP53-RNA was purified as previously described (Liang et al., 2013). The collected cells were digested with mutanolysin (500 units/ml) in 300 μl of spheroplasting buffer (20 mM Tris–HCl-10 mM MgCl_2_/56% raffinose, and pH 6.8) and then incubated with 100 mg/ml chloramphenicol at 37°C for 60 min. The cell pellets were harvested by centrifugation at 10,000 rpm for 5 min. Total RNA was isolated and purified from the cell pellets using a DNeasy blood and tissue kit (Qiagen, Valencia, CA, United States). The RNA collected was treated with DNase I twice to efficiently remove genomic contaminations and elution was performed with nuclease-free water. The quality of the extracted final RNA was assessed by the A_260_/A_280_ ratio (~1.1) and visualized in 1% agarose.

Two or three independent extractions of total RNA from each of the strains were used for rt-PCR. To detect *sen* gene transcription, a 23 bp forward primer beginning at nucleotide 604 and a 24 bp reverse primer beginning at nucleotide 823 was used to yield an amplicon of 220 bp. The relative gene expression levels were analyzed by the 2^−ΔΔCt^ method, in which Ct represents the threshold cycle number of RT-PCR at which the amplified product was first detected. The statistical means of triplicate Ct values were calculated for the target and GAPDH as reference genes from both WT and mutant strains as previously described (Liang et al., 2013).

### Subcellular Localization of Sen in GAS Cells by Western Blotting

Single colonies of AP53 cells were grown overnight in THY-5% CO_2_ at 37°C. Aliquots of 5 ml were inoculated into 45 ml of pre-warmed THY medium. The colonies were grown to mid-log phase. After centrifugation, the supernates were separated from the cells and the cells were treated with PlyC. The digests were separated into cell wall (CW), cell membrane (CM), and cytoplasm (CP) fractions as described earlier (Russo et al., 2020). Each cell fraction was mixed with SDS-PAGE loading buffer and boiled for 8 min. Aliquots (10 ml) of each sample, along with 25 ng of rSen, were loaded onto 12% SDS-PAGE gels and used for Western blotting. Rabbit-anti Sen was used as the 1^o^ antibody and goat-anti-rabbit IgG-HRP was used as the 2^o^ antibody. The results were then developed using luminol-peroxidase (Thermo-Fisher).

### Biotinylation of Whole Cells

Accessible proteins of AP53, AP53/*Δpam*, and AP53/*pam[8A]* cells were biotinylated, and the biotin-labeled proteins were isolated as described (Bonn et al., 2018). For this, cells at the mid-log growth phase were washed twice in PBS/1 mM phenylmethylsulfonyl fluoride (PMSF), pH 8.0, and resuspended in the same buffer. Sulfo-N-hydroxysulfosuccinimide (NHS)-SS-biotin, dissolved in PBS, was added to the cell suspensions to final concentration of ~0.9 mg/ml. The cells were incubated for 1 h on ice with gentle rotation at 4°C, after which the reaction was terminated by centrifugation for 1 min at 20,000 *g*. The supernate was removed and the cells were washed 3x with PBS/0.5 M glycine after which they resuspended in PBS/5% iodoacetamide.

To isolate the CW, the cells were washed 3x with PBS. Following this, the cells were subjected to PlyC digestion and ultracentrifugation (Russo et al., 2020). Biotinylated proteins from the CW fractions were isolated by incubation in microcentrifuge tubes of the CW fractions with 150 μl streptavidin-agarose, pre-equilibrated with PBS, pH 8.0. The tubes were then centrifuged to remove the supernatant, and the resin was washed 3x with 1 ml PBS to remove unbound proteins. Bound proteins were eluted by incubating the resin with 5% β-mercaptoethanol in water for 1 h at 25°C. The tubes were centrifuged and the supernatant was added to 8 ml of pre-chilled acetone. The tubes were then washed with another 1 ml of the elution buffer, centrifuged, and the supernatant added to the pre-chilled acetone overnight at −20°C. On the next day, acetone precipitates were recovered by centrifugation at 10,000 *g* at 4^o^C. The precipitates were washed in 1 ml pre-chilled ethanol, centrifuged at 15,000 *g* at 4°C to remove the ethanol, after which the precipitates were vacuum dried at 30°C and resuspended in PBS. A control experiment was similarly performed with CW fractions isolated from non-biotinylated bacterial cells.

The samples were analyzed for the presence of Sen by Western blot analysis as described above. Control blots were analyzed for the presence of PAM as a positive control. Western blotting to detect PAM was performed as described for Sen, except that the primary antibody was replaced by a polyclonal antibody raised against PAM.

### Scanning Electron Microscopy of Isogenic AP53 and SF370 Isogenic Cells

The methods used were adapted from the collection of techniques for SEM sample preparation (Fischer et al., 2012). Specifically, mid-log growth phase cells from 40 ml cultures were centrifuged and the pelleted cells were washed 3x with PBS. The cells were then incubated at room temperature with rabbit-anti Sen and/or hPg (400 nM) followed by mouse anti-hPg in 10 ml PBS. Negative controls were incubated in PBS without added antibody. Next, the cells were centrifuged and washed 2x with PBS. The cell pellets were placed in 1 ml of 2% glutaraldehyde/0.1 M sodium cacodylate/PBS as the first fixative solution. Two μl of the dispersed pellets were pipetted onto glass microscope slides coated with 0.01% poly-L-lysine and allowed to remain for 1 h for cross-linking to occur. These samples were next placed in 1 ml 1% osmium tetroxide, and then washed 3x with PBS. Water was removed from the samples by sequentially adding 50, 70, 80, 95%, and 3× 100% ethanol (3x). The ethanol was then removed, and liquid CO_2_ was added to remove any excess ethanol. The slides were then attached to metal Scanning Electron Microscopy (SEM) stubs pre-coated with a 3 nm gold coating using a sputter coater. The samples were then imaged under conditions of negative-charging contrast at 80,000x at 1 KeV using a Magellan 400 FESEM. All samples prepared at the same time and were under identical conditions.

### Statistical Analysis

Statistical analyses were performed using GraphPad Prism 9.0. Error values were expressed as mean ± SD of at least three biological or experimental replicates. The data were statistically compared using one-way analysis of variance (ANOVA) with Dunnett’s T3 multiple comparisons test. Probability values (*p*-values) considered statistically significant are indicated by asterisk(s), as follows: *p < 0.05; **p < 0.01; ***p < 0.001; and ****p < 0.0001.

## Results

### Characterization of Materials

**Figure 1:**
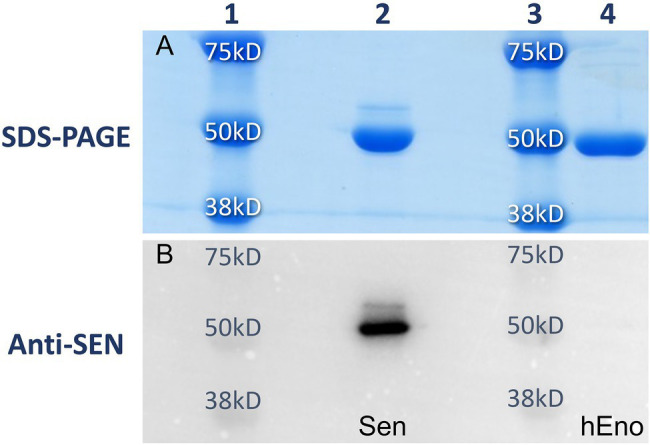
Purities of recombinant Sen and hEno. Recombinant Sen (lane 2) and hEno (lane 4) monomeric molecular weights and purities as determined using 12% SDS-PAGE **(A)** and Western blotting with rabbit-anti Sen **(B)**. With 67% amino acid sequence similarities between these proteins, no cross-reactivity of anti Sen was observed with hEno. Lanes 1 and 3 are molecular weight standards.

The expression and purification of streptococcal enolase (Sen) and human enolase (hEno) from *E. coli* cells provided ample amounts of purified materials (>50 mg/L). The proteins were isolated from transformed lysed cells and purified using Ni^+^-agarose affinity chromatography. The high quality of the purified proteins was demonstrated by SDS gels stained with Coomassie blue (Figure 1A), showing single subunit bands in the proper range of ~49,000 Da for recombinant Sen and hEno, as expected. In addition, Western blot analysis with anti-Sen polyclonal antibodies generated in rabbits showed a corresponding band at the location of Sen and did not cross-react with hEno (Figure 1B).

**Figure 2:**
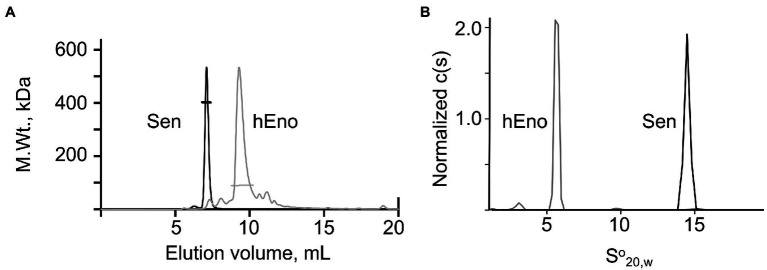
Characterization of Sen and hEno. Quaternary structures of Sen and hEno. **(A)** Molecular weights of Sen and hEno by Size Exclusion Chromatography (SEC)-multi-angle light scattering (MALS). Each protein (100 μl) was separately injected onto a Wyatt—030S5 column (7.6 mm × 300 mm, 5 μM, 300 Å), equilibrated and eluted with PBS, pH 7.4, at a flow rate of 0.5 ml/min. The flow through was passed in-line through a three-angle light scatter apparatus coupled to a refractive index (RI) detector. RI fractograms for Sen (black line) and hEno (gray line) are plotted as M.Wt. vs. elution volume. The bars across each peak indicate the values of the molecular weights. The M.Wt. of the Sen is consistent with an octameric structure, while hEno is a dimeric structure. **(B)** Normalized sedimentation coefficient distributions of Sen and hEno at 20°C were obtained at 0.5, 1.0, and 2.0 mM for Sen and 6, 11, and 21 mM hEno (based on the M.Wts. of the Sen octamer and hEno dimer). The rotor speed was 30,000 rpm for Sen and 35,000 rpm for hEno, and the buffer was 50 mM Na-phosphate/0.1 M NaCl, pH 7.4. The data are plotted as the normalized continuous distributions, c(s), against the S^o^_20,w_ which were obtained by fitting the raw data to the Lamm equation using SEDFIT (available at https://spsrch.cit.nih.gov/). Scans were obtained every 3 min for 16 h.

The quaternary structures of Sen and hEno were determined by SEC-MALS (Figure 2A) and AUC (Figure 2B) in native buffers. Mammalian enolases consist of a, b, and g isoforms that can exist as homo- or hetero-dimers, depending on the tissue source (Tracy and Hedges, 2000). By SEC-MALS, a Mol. Wt. of 92,000 ± 1,500 for hEno was obtained. AUC experiments provided a Mol. Wt. of 91,800 ± 2,000 and a S^o^_20,w_ value of 5.8 ± 0.1S for hEno, all consistent with a dimeric structure. On the other hand, using SEC-MALS, Sen showed a Mol. Wt. of 393,800 ± 8,000 and a S^o^_20,w_ value of 14.5 ± 0.2S, thus representing an octameric structure (Ehinger et al., 2004). We found no evidence of concentration-dependent dissociation of Sen or hEno in solution as neither Sen nor hEno showed Mol. Wt. or S^o^_20,w_ values that changed as their concentrations were decreased to at least 0.1 mg/ml. This demonstrated that their quaternary structures were stable dimers and octamers, respectively.

To further characterize Sen and hEno, we compared their enzymatic activities in the conversion of 2-phosphoglycerate to phosphoenolpyruvate, with 10 mM MgCl_2_, at 240 nm (Wold, 1971). The activities were similar at 0.151 ± 0.008 and 0.126 ± 0.007 µmol pyruvate/min/µg of enolase, respectively.

### Binding and Activation of hPg to Sen and PAM in Solution

**Figure 3:**
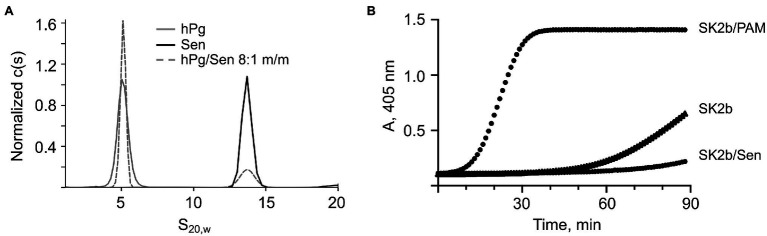
Interaction of hPg with Sen in solution. **(A)** Sedimentation velocity analysis of the binding of hPg to Sen. An 8:1 m/m mixture of hPg (0.8 μM) and Sen (0.1 μM) were mixed and subjected to sedimentation velocity analysis. The two peaks (dashed lines) obtained, shown as overlay of individual sedimentation coefficient profiles of hPg and Sen (solid lines), corresponded to free hPg and free Sen (solid lines) verifying their lack of interaction in solution. Sedimentation velocity experiments were performed at 35,000 rpm at 20°C, in 50 mM Na_2_HPO_4_/100 mM NaCl, pH 7.4. The peaks were overlaid using GUSSI https://www.utsouthwestern.edu/labs/mbr/software/. **(B)** Activation of hPg by SK2b in solution. A mixture of hPg (200 nM) and Sen (0 or 8 μM) or PAM (0.25 μM) were added to the wells of non-protein binding microtiter plates and incubated for 10 min at 25°C. The chromogenic substrate, S2251 (0.25 mM), was added and the activation was accelerated by addition of SK2b (5 nM). The release of p-nitroaniline from S2251 was continuously monitored for 90 min at A_405nm_.

Our main goal in this study was to evaluate the importance of Sen in hPg binding and activation, especially in GAS cells that also contain a direct hPg binding M-protein (PAM), a characteristic of Pattern D GAS. PAM tightly binds and greatly stimulates the activation of hPg by the coinherited SK subform, SK2b, in solution and on the GAS cell surface (Zhang et al., 2012, 2014). On the other hand, sedimentation velocity studies demonstrate that Sen and hPg do not interact when present in a mixture of 8:1 m:m of hPg:Sen (Figure 3A). Both hPg and Sen retained their characteristic S^0^_20,w_ values in the mixture, clearly showing a lack of interaction under these conditions. In addition, ITC studies (not shown) demonstrate that there was no significant heat change when hPg was titrated into Sen, or vice versa. These data demonstrate that Sen does not meaningfully bind to hPg in solution, whereas the Kd of the hPg/PAM complex in solution is ~1 nM. Coupled with this finding are experiments that demonstrate that Sen does not stimulate hPg activation by SK2b in solution, whereas PAM shows a high level of stimulation toward SK2b at very low concentrations (Figure 3B).

### Binding and Activation of hPg to Sen on Artificial Surfaces

**Figure 4:**
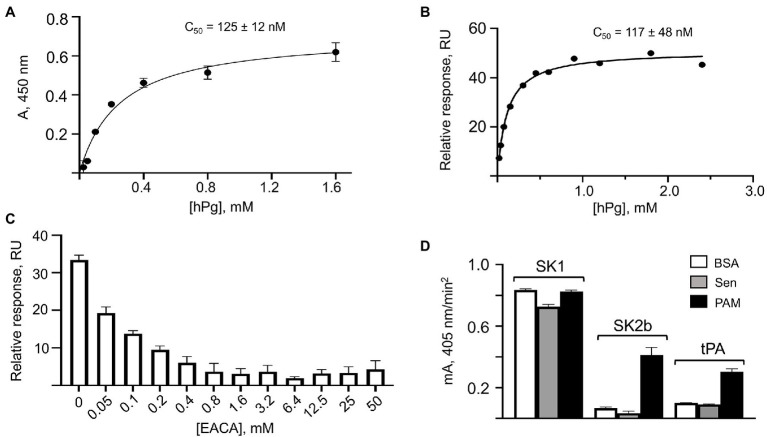
Binding of hPg to Sen on surfaces. **(A)** Interaction of hPg with plate-bound Sen by ELISA. Sen was bound to individual wells of a 96-well high protein binding microtiter plates. Next, hPg was added, followed by HRP-anti-hPg. HRP activity was detected using the substrate, TMB/H_2_O_2_. The A_280 nm_ is plotted against [hPg], thereby allowing determination of the C50 from the fitted curve. The saturation curve and C_50_ value for the binding of hPg to Sen were generated using GraphPad Prism 9.0. **(B)** Binding of hPg to chip-immobilized Sen by surface plasmon resonance (SPR). The response units (RU) as a function of hPg analyte concentrations were fit using the equilibrium method allowing the determination of the C_50_ of binding. **(C)** e-aminocaproic acid (EACA) competition for the binding of hPg to Sen by SPR. Sen was bound to the chip and hPg (1 μM) analyte in the presence of the indicated EACA concentrations was flowed through the chip surface. The RU vs. [EACA] is shown in the plot. **(D)** Stimulation of the activation of hPg by Sen. Multiple wells of a high protein binding microtiter plate were coated overnight with Sen, PAM, or BSA. hPg was added to the wells and activation of hPg was initiated by addition of catalytic levels of SK1, SK2b, or tPA, each mixed with the chromogenic substrate, S2251 (H-D-Val-Leu-Lys-pNA). The release of p-nitroaniline from S2251 was continuously monitored for 120 min at A_405nm._ The initial rates of activation were obtained from the linear regions of the A_405nm_ vs. time plots and shown as the mA_405nm_/min^2^.

While hPg and Sen do not interact to a biologically relevant extent in solution, the binding of these components is measurable when Sen is bound to a surface. Sen, bound to wells of a 96-well high protein binding microtiter plate, followed by addition of various concentrations hPg and HRP-anti-hPg, displayed a titration curve using ELISA methodology (Figure 4A). This allows the C_50_ of the interaction to be estimated to be ~125 nM. Similarly, when Sen was bound to a surface plasmon resonance (SPR) chip, and various concentrations of hPg flowed over the chip until a steady state RU was reached, the data of Figure 4B were obtained. In this case, a very similar C_50_ of 111 nM was calculated, a value very close to that found for plate-bound Sen. This interaction is dependent on the LBS of hPg as shown by the displacement of hPg with ε aminocaproic acid (EACA; Figure 4C), a lysine analogue, with a half-maximal concentration of ~0.1 mM. This value is consistent with the strength of interaction of EACA with kringle domains of hPg (Sehl and Castellino, 1990).

We next evaluated the ability of surface-bound Sen to stimulate hPg activation by hPg activators (Figure 4D). Here, we note that SK1 (a subform of streptokinase found in GAS strains that do not contain PAM-type M-protein) does not discriminate between hPg bound to Sen or PAM, or hPg free in solution, as we have indicated previously (Zhang et al., 2012). With SK2b as the activator, which requires hPg to be bound to its receptor (PAM) to be activated, there is no difference in activation rates between free hPg (BSA-coated plate) and Sen-bound hPg on Sen-coated plates. However, the activation rate of hPg by SK2b is highly stimulated when hPg is bound to PAM, confirming earlier results (Zhang et al., 2012). Similar observations apply when tPA is used as the activator of hPg (Figure 4D). In this case, the rate of activation of hPg bound to Sen was not stimulated by tPA when compared to free hPg, whereas the tPA-catalyzed activation of hPg bound to PAM shows a ~2.5-fold rate stimulation under the same conditions. We conclude that while Sen bound to artificial surfaces interacts with hPg, this interaction is not functional with regard to stimulation of the rate of activation of hPg.

### Binding of hPg/Sen to Phospholipid Vesicles

**Figure 5:**
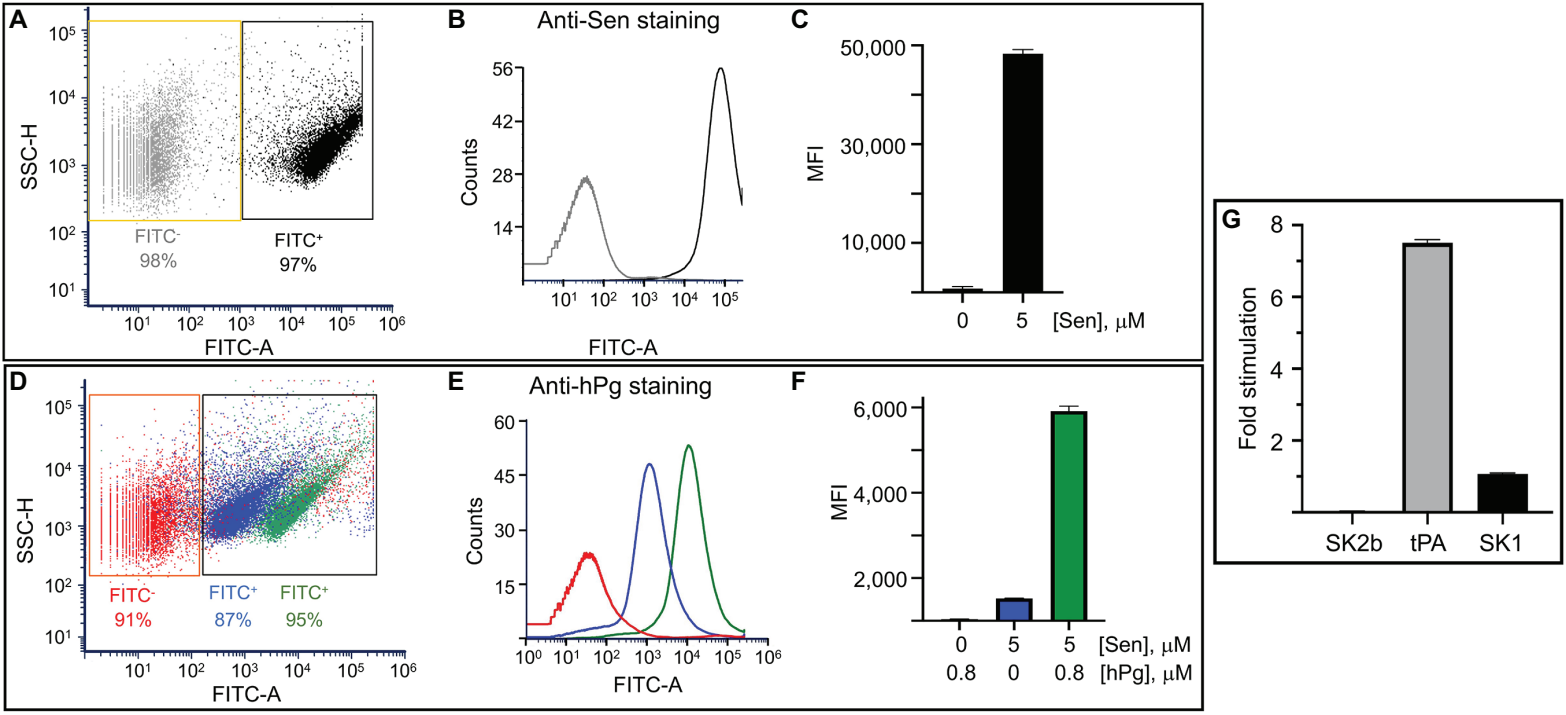
The interaction of Sen with DOPG vesicles as measured by flow cytometric analysis (FCA). **(A)** Dot plot overlay of DOPG vesicles incubated with Sen (0-gray or 5 μM-black) followed by rabbit anti-Sen and FITC-chicken-anti-rabbit IgG. The plot shows the side scatter of DOPG vs. FITC-derived fluorescence and the FITC-based gates that define Sen-bound and non-bound DOPG vesicles. **(B)** Histogram overlay of DOPG from A vs. FITC intensity. **(C)** Bar representation of median fluorescence intensity (MFI) obtained from B. **(D)** Dot plot overlay of DOPG vesicles incubated with Sen (0- red or 5 mM—blue and green) followed by hPg (0—blue or 0.8 μM—red and green), then mouse anti-hPg and FITC-donkey-anti-mouse IgG. The plot shows the side scatter of DOPG against FITC-derived fluorescence and the FITC-based gates that define IgG-bound and non-bound DOPG vesicles. **(E)** Histogram overlay of DOPG from D vs FITC intensity. **(F)** Bar representation of median fluorescence intensity obtained from E. **(G)** Stimulatory effect of PL-bound Sen on the activity of hPg activators. PL vesicles (0.1 mM) pre-blocked in 2.5% BSA were subjected to 1 h incubation in 0 or 5 μM Sen. The PL was washed, resuspended in PBS, and added in replicates to wells of a non-protein binding 96-well microtiter plate. hPg (200 nM, final; concentration) was next added, after which a mixture of chromogenic substrate, S2251 (H-D-Val-Leu-Lys-pNA; 0.25 mM) admixed with SK2b, tPA, or SK1 (5 μM). The reaction was continuously monitored for 120 min at A_405nm_. The fold increase in reaction velocity was calculated by dividing the initial velocities of assays performed in PL-Sen (5 μM) by those performed in PL-Sen (0).

Since Sen bound to surfaces is more efficient at binding hPg than Sen in solution, we next assessed whether Sen interacted with PL vesicles as a mimetic of membrane-bound Sen. For this experiment, we prepared small vesicles using 1,2-dioleoyl-s:n-glycero-3-[phospho-rac-(1-glycerol)] (DOPG) as the PL. FACS analysis (Figures 5A–C) clearly showed that Sen was bound to PL and that hPg interacted with PL-bound Sen at the μM level (Figures 5D–F). Thus, the PL vesicle provides for Sen an orientation that optimally juxtaposes its binding site with that of hPg.

The next question is whether the activation of hPg bound to PL surfaces becomes more facile. The data of Figure 5G show once again that when SK1 and SK2b are used as activators, there is no stimulation of hPg when bound to the PL matrix. However, when tPA was used as the hPg activator, a 6–8 fold stimulation of the rate of hPg activation was observed.

### Binding and Activation of hPg on Isogenic GAS Cell Lines

**Figure 6:**
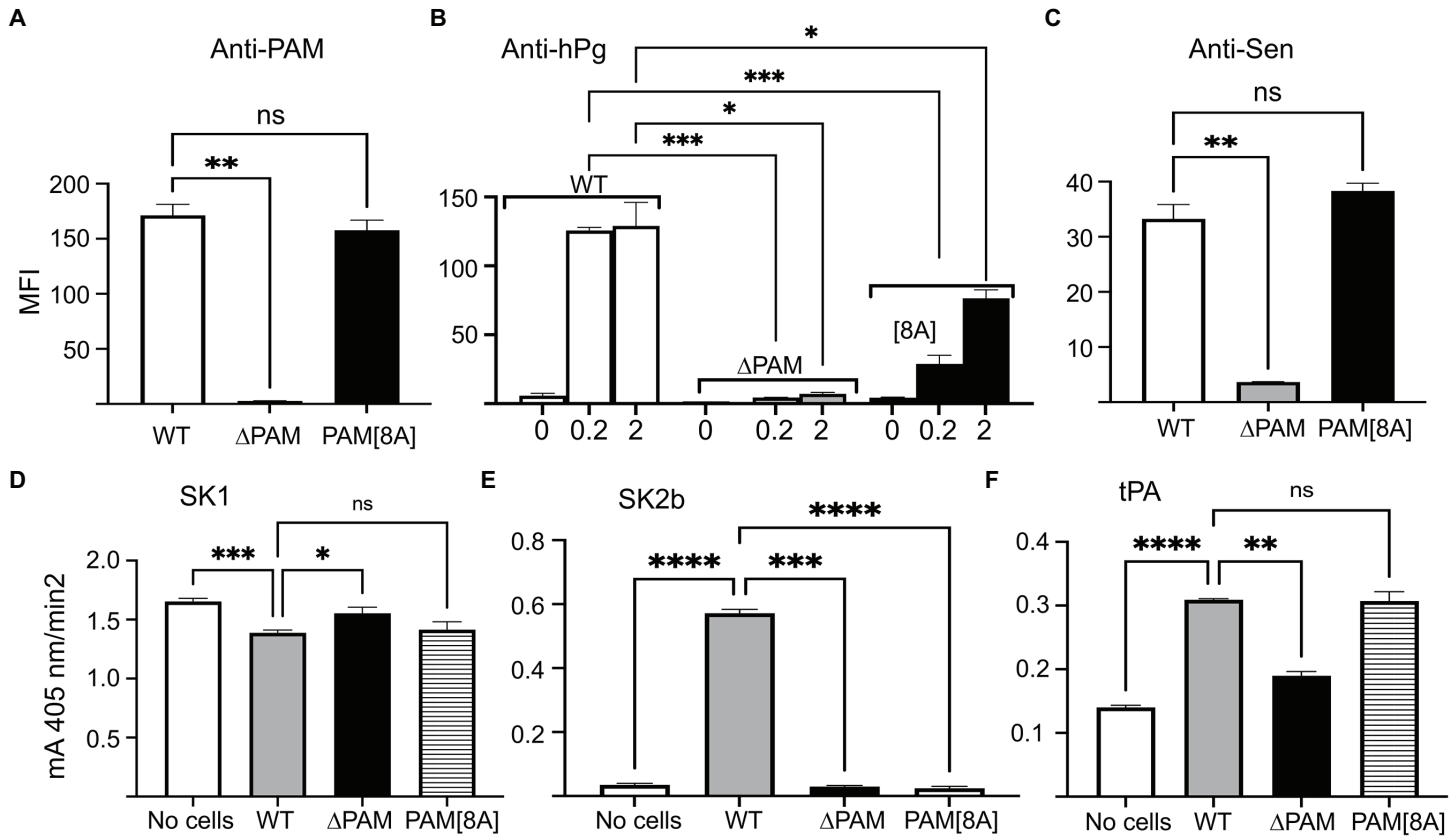
Binding and activation of hPg on whole GAS cells. Mid-log growth phase WT-AP53, AP53/*Δpam*, and AP53/*pam[8A]* cells were incubated with: **(A)** polyclonal rabbit-anti PAM; **(B)** monoclonal mouse-anti hPg after addition of 200 nM hPg to the cells, and **(C)** polyclonal rabbit-anti Sen. After this, Alexa Fluor 488-chicken-anti-rabbit IgG was added to cells in **(A,C)** and Alexa Fluor 488-donkey-anti-mouse IgG for cells of **(B)**. The fluorescence intensity data were acquired by FACS and reported in bar graph format as the median fluorescence intensity (MFI) of the respective isogenic strains. **(D–F)** Effect of GAS cells on hPg activation by plasminogen activators. The indicated isogenic mid-log phase AP53 cells (CFU ~ 2 × 10^8^) were blocked with PBS/1%BSA and 50 μl of each cell suspension was added to individual wells of 96-well non-protein binding microtiter plates, after which hPg was added to a final concentration of 200 nM. hPg activation was initiated by the addition of 5 nM of **(D)** SK1, **(E)** SK2b, or **(F)** tPA admixed with S2251 (0.25 mM). The release of p-nitroaniline (A_405nm_) from the chromogenic substrate was continuously monitored for 120 min. The initial rates of activation were calculated from the linear regions of A_405nm_ vs. time plots and shown as the mA_405nm_/min^2^. The statistical analyses of data for WT-AP53 cells compared with those of AP53/*Δpam* cells and AP53/PAM[8A] provided probability (*p*) values as indicated by asterisk(s). *p < 0.05; **p < 0.01; ***p < 0.001; ****p < 0.0001; ns, not significant.

To measure the binding of hPg to GAS cells that contain both PAM and Sen, we compared the binding of hPg to WT-AP53 cells and isogenic AP53 cells with a targeted gene deletion of the *pam* gene (AP53/*∆pam*). As expected, neither anti-PAM (Figure 6A) nor anti-hPg (Figure 6B), interacted with *pam*-ablated cells. Stimulation of hPg activation by SK1 (Figure 6D), SK2b (Figure 6E), or tPA (Figure 6F) did not occur in cells lacking PAM, but presumably containing Sen. Based on these results it seems straightforward to conclude that Sen does not play a role in hPg binding or activation of hPg in GAS cells that also contain PAM. However, additional proof is needed since studies with anti-Sen binding to GAS cells show that expression of Sen on the GAS cell surface is also greatly diminished in cells with a targeted deletion of the *pam* gene (Figure 6C). To address this issue, we generated an isogenic GAS-AP53 cell line with eight amino acid substitutions (AP53/PAM[8A]) in the PAM A-domain that eliminated hPg binding in an otherwise intact PAM (Bhattacharya et al., 2014). This variant cell line showed full PAM expression (Figure 6A) and restored full cell surface expression of the Sen epitope (Figure 6C). Importantly, hPg binding did occur with the AP53/*pam[8A]* cells (Figure 6B), suggesting that other hPg direct binding proteins were present on the GAS cell surface and interacted with hPg. Nonetheless, despite binding hPg to some degree, there was no stimulation of hPg activation by SK1 (Figure 6D) or SK2b (Figure 6E). Activation with tPA, however, displayed a low degree of stimulation similar to WT-AP53 cells (Figure 6F). This further suggests that functional Sen was restored on the cell surface. We conclude that proteins other than PAM are not functional in stimulating the activation of hPg by GAS secreted activators on the GAS cell surface, but host activators, e.g., tPA, can offer some small degree of stimulation.

### SEM Analysis of the Presence of Sen on the Cell Surface of Isogenic AP53 Cells

SEM analysis of cells obtained by use of secondary electrons combined with negative charging contrast (Zhang et al., 2003, 2004) in which cells were immunolabeled with anti-Sen have been performed as qualitative support of the main conclusions of this manuscript. Using this approach, the main conclusions of Figures 6A–C are confirmed by the SEM micrographs of Supplementary Figure 1. Here, mid-log GAS cells are shown without added antibody (Supplementary Figure 1A) as compared to WT-AP53 cells treated with rabbit-anti Sen (Supplementary Figure 1B). This shows that Sen is abundant on the surface of these cells and that hPg binds to WT-AP53 cells when hPg was added and then exposed to rabbit-anti-hPg (Supplementary Figure 1C). However, in cells in which the *pam* gene has been deleted (AP53/*∆pam*), there is no observable Sen on the cell surface (Supplementary Figure 1D). This shows that surface expression of Sen, directly or indirectly, is dependent on PAM expression. Lastly, when PAM is replaced by a targeted PAM variant (AP53/PAM[8A]) that does not bind hPg, full expression of Sen is observed (Supplementary Figure 1E). This demonstrates that the presence of Sen on the cell surface is dependent on PAM. Yet when Sen is fully restored on cells expressing PAM[8A] variant, hPg only minimally interacts with Sen (Supplementary Figure 1E). This further demonstrates that PAM is the most dominant receptor of hPg on the GAS cell surface.

To show that the codependency of PAM and Sen expression on the GAS cell surface is not a singular property of AP53 cells, we also examined this issue employing a very different GAS strain, SF370, that expresses the *emm1* (M1) M-protein, instead of PAM. M1-protein does not directly interact with hPg (Chandrahas et al., 2015). The data obtained (Supplementary Figure 2) are very similar to those data seen using isogenic GAS-AP53 variants. A similar Sen epitope is expressed on the surface of these cells (Supplementary Figure 2A) and Sen is not found on the cell surface when the M1 protein is deleted (SF370/∆M1; Supplementary Figure 2B). The FCA data (Supplementary Figure 2C) are similar to those of SEM and also show the greatly attenuated distribution of Sen on the GAS cell surface in the absence of the M1 protein. Thus, the codependency of Sen and M-protein on the cell surface is verified using another strain of GAS.

### Subcellular Distribution of Sen in Isogenic AP53 Cells

**Figure 7:**
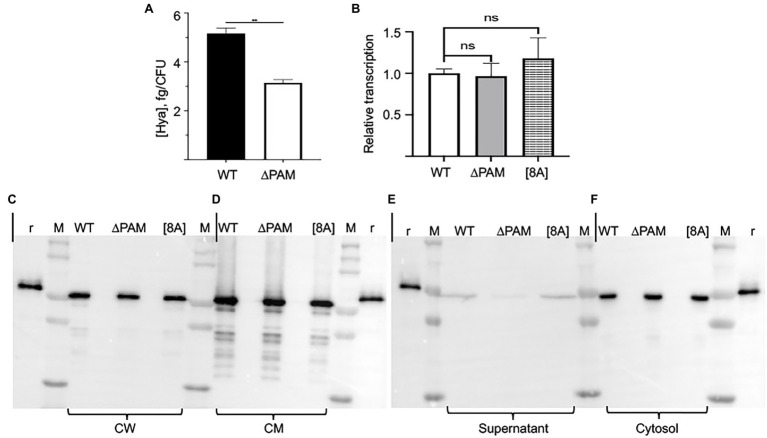
Expression of Sen in subcellular compartments of AP53, AP53/ΔPAM, and AP53/*pam[8A]* cells. **(A)** Quantitation of the capsule in WT-AP53 and AP53/*Δpam* isogenic cells. Mid-log cells were separated in two fractions, one for determination of the CFU and the other for determination of hyaluronic acid (Hya). Hya standards were also employed. The results are expressed as fg Hya/CFU. **(B)** Transcription of Sen in isogenic AP53 cell lines. Total RNA from mid-log phase AP53, AP53/*Δpam*, and AP53/*pam[8A]* isogenic strains were used to quantitate *sen* gene transcription using the same primers as for RT-PCR. The relative gene expression levels were analyzed by the 2^−ΔΔ*Ct*^ method. The statistical means of triplicate Ct values were calculated. GAPDH was used as the reference gene for both WT and mutant strains. Expression of Sen in mid-log phase cells of various genotypes: **(C)** cell supernatants (15x concentrated); **(D)** cell walls (CW); **(E)** cell membranes (CM); **(F)**, cell cytosols. In all cases: r, recombinant Sen; M, molecular weight marker; WT-AP53 cells (WT); AP53/*∆pam* cells (∆PAM); AP53/*pam[8A]* (8A).

Since Sen is not present on the GAS cell surface when the *pam* gene is deleted, we first assessed whether a *pam* deletion affected the transcription of *sen*. The data of Figure 7B show that when compared to WT AP53 cells, the transcription of Sen is not altered by deletion of the *pam* gene in AP53 cells or expression of the PAM[8A] variant. Further, the level of outer capsule is diminished in AP53/∆pam cells, as compared to WT-AP53 cells (Figure 7A). This is an important consideration since it was possible that an increase in capsule in AP53/∆pam cells could screen Sen present in the CW, but that is clearly not the case.

We next examined the subcellular distribution of Sen in various isogenic AP53 cell lines (Figures 7C–F). While Sen is present in cell wall (CW; Figure 7C), cell membrane (CM; Figure 7D), and cytosolic (Figure 7F) fractions of WT-AP53, AP53/*∆pam*, and AP53/*pam[8A]* cells, very little is released into the cell supernatants (Figure 7E) of mid-log phase AP53 cells. Thus, Sen is present in each of the cell subfractions in all three isogenic strains of GAS in very similar amounts in each of the strains.

These data are particularly relevant for AP53/*∆pam* cells. While the presence of Sen in the cell supernates, cytoplasm, and CM, is clear, its presence in the CWs is open to some level of concern. To obtain the CW fraction, digestion of isolated spheroplasts with PlyC is employed with the reaction conducted over a period of time (1 h in our case). Thus, it is possible that during this digestion period, Sen could leak from the cytoplasm or CM and contaminate the CW fraction. We have attempted to resolve this matter by varying the amounts of PlyC and digestion times and still find Sen in all the CW fractions. Thus, a portion of the expressed Sen appears to reside in the CW, likely due to noncovalent binding with components such as CW techoic acids.

**Figure 8:**
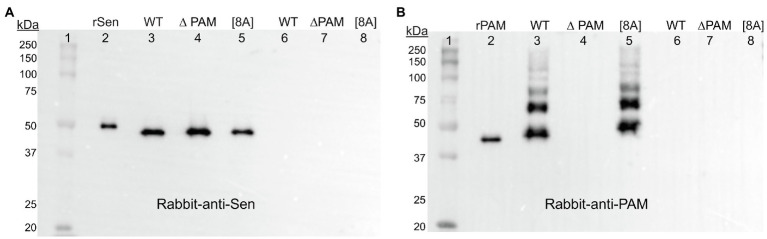
Presence of biotinylated AP53 proteins in the CWs of mutant strains of AP53 GAS. The isogenic AP53 strains were incubated with sulfo-NHS-SS-biotin prior to PlyC treatment and excess biotin washed from the cells. PlyC was then added and the CW digests containing biotin-labeled proteins were isolated by streptavidin-Sepharose columns. Biotinylated proteins were eluted with mercaptoethanol. The samples were analyzed by: **(A)** Western blotting with rabbit anti-Sen. Lane 1, molecular weight markers; Lane 2, rSen; Lane 3, WT-AP53 (WT); Lane 4, AP53/∆*pam* (∆PAM); Lane 5, AP53/*pam[8A]* [8A] cells. Lanes 6–8 are cells not treated with sulfo-SS-NHS-biotin and used as negative controls for samples in Lanes 3–5. **(B)** As in **(A)** except that rabbit-anti-PAM was used and Lane 1 is rPAM in place of rSen.

A further step to more unequivocally answer this question involved biotinylation of the CW proteins in intact AP53 cells prior to PlyC digestion. Sulfo-NHS-SS-biotin, which does not cross the CM, was added to the AP53-WT, AP53*/∆pam*, and AP53/*pam[8A]* cells prior to PlyC digestion. After removal of excess biotin by washing, the AP53 cells were digested with PlyC, and the biotinylated CW fractions were isolated by binding to a column of Sepharose-streptavidin. Subsequent to washing the column with buffer, the biotinylated proteins were eluted by cleavage of the S-S bond with mercaptoethanol, and the eluates were examined by Western analysis with rabbit-anti Sen (Figure 8A) and rabbit-anti PAM (Figure 8B). The results showed the presence of Sen in the CWs of AP53-WT, AP53*/∆pam*, and AP53/*pam[8A]* (Figure 8A) strains. As a positive control, PAM was also found in equivalent amounts and covalently bound to the CWs of WT-AP53 cells and AP53/*pam[8A]* cells, but not in AP53/∆*pam* cells (Figure 8B). Overall, a most interesting point is that while Sen is present in the CW in AP53/∆*pam* cells, it is no longer exposed on the cell surface to either anti-Sen or to hPg. Thus, the expression of Sen on the cell surface is linked to PAM expression.

## Discussion

Enolase is a cytoplasmic metallic hydrolyase that reversibly catalyzes the penultimate ninth step of the glycolytic pathway, yielding phosphoenolpyruvate. Its well-established role in glycolysis in the cytoplasm of cells is its major enzymatic function. However, Eno is also ubiquitously present on cell surfaces where it displays non-enzymatic moonlighting properties. The mechanism of the trafficking of Eno from the cytosol to the cell membrane is not fully understood. Eno does not possess a signal sequence, indicating that Eno does not employ the classical ER-Golgi apparatus for secretion from the cytosol. Eno also does not contain any known primary sequences that target this protein to specific known locations in the cell, except that, at least in yeast, it has been shown that the N-terminal 169 residues of Eno is sufficient for its export out of the cytoplasm and to the cell surface (Lopez-Villar et al., 2006). Several mechanisms have been proposed for unconventional transport of leaderless proteins (Cohen et al., 2020), such as plasma membrane blebbing, fusion of cargo-containing vesicles with the cytoplasmic membrane assisted by SNARE-dependent proteins (Miura et al., 2012), and other transporters in the plasma membrane (Andrei et al., 1999). In fact, exosomes containing Eno have been found outside of the cytoplasm (Salvia et al., 2019), suggesting this as a possible method for the ability of Sen to cross the cytosolic membrane.

It is well established that Eno (or Sen in the case of GAS) is present on the outer surfaces of cells. Surface-exposed Eno or Sen is an important cofactor in generating proteolytic activity on cellular surfaces, thus enabling cells to migrate and disseminate, serving processes such as inflammation, extracellular remodeling, and invasion, with functional ramifications in many physiological and pathological states (Miles et al., 1991). The major protease on such cell surfaces is host hPm, which arises from activation of hPg, hijacked to the cell surface by hPg binding proteins, e.g., Eno. For this function to be important, the adsorbed hPm must be protected from inhibition by the natural hPm inhibitor, a_2_-antiplasmin, in order that a stable protease can function on cells. These criteria are met with Eno on the surface of bacteria (Miles et al., 1991). However, further considerations are needed with Gram-positive bacteria, such as GAS, which do not possess a cell outer membrane to stabilize Sen. Nonetheless, we show that Sen is present in all subcellular fractions of GAS and is exposed on the cell surface. It appears that Sen escapes the cytoplasm, is present on the cytosolic membrane and bound to the CW where it exposes important epitopes for hPg binding. To prove that Sen is bound within the CW, and is not an artifact from the cell subfractionation procedures, it is shown herein by experiments in which the GAS cells were treated with sulfo-SS-NHS-biotin, prior to isolation of the cell subfractions, that biotinylated Sen was found in ample amounts in the CW fraction.

A most interesting observation is that the expression of Sen on the cell surface is dependent on M-protein expression. Sen does not show surface expression in AP53/∆*pam* cells, or in an unrelated strain of GAS, SF370/∆*M1* (*emm1*) cells, either through lack of anti-Sen binding to AP53/∆*pam* cells or through SEM studies of these variants. This is not due to effects of transcription of PAM on Sen. We show that Sen is present in the CWs of AP53/∆*pam* cells in amounts very similar to the WT-AP53 cells, but the protein is not exposed on the cell surface. This issue is unlikely of importance to Sen trafficking since Sen is reactive to its antibodies in the CW of both WT-AP53 cells and AP53/∆*pam* cells. A logical explanation is that the absence of PAM on the CW rearranges CW components in a manner that binds Sen differently, leading to its lack of exposure on the cell surface. A complementation-type experiment in which a mutant form of PM was engineered in a targeted fashion into AP53/∆*pam* cells, resulting in expression of AP53/PAM[8A] (which does not bind to hPg), completely restores Sen surface exposure perhaps by reconstructing the proper Sen binding sites in the CW.

While Eno appears to be an important hPg constituent of the surface of many types of cells, our initial question concerned whether Sen was important as a hPg receptor in the abundant lines of Pattern D GAS cells, which expressed a M-protein (PAM) with a very high affinity for hPg. The initial, presumably simple, experiment of measuring hPg binding to GAS with a targeted deletion of PAM indeed showed a lack of hPg binding. This could lead to the conclusion that Sen was not important to hPg binding to Pattern D GAS cells. However, we then showed that Sen was also not surface exposed in AP53/∆PAM cells or in cells with an M-protein deletion that was unrelated (McNeilly and McMillan, 2014) to Pattern D GAS cells. These experiments were then conducted on GAS cells with a targeted replacement of PAM, albeit mutated to be unreactive with hPg. In this case, surface binding of hPg that was much weaker than hPg binding to PAM, occurred which likely was related to Sen re-exposure. However, hPg bound to the cells *via* proteins unrelated to PAM was not activated by the coinherited SK2b subform of SK, which is the resident SK in all Pattern D strains of GAS. The data also show that the major receptor for hPg is PAM, when present, which tightly binds to hPg and stimulates its activation by the coexpressed SK2b. The activation of hPg bound to PAM and to Sen is slightly stimulated toward activation by host tPA, but much less so than stimulation of hPg bound to PAM by SK2b, demonstrating that PAM is the major functional receptor in these classes of GAS.

In conclusion, we show that PAM is the dominant receptor for hPg in GAS cells that contain this tight binding PAM-like M-protein. We further conclude that the weaker binding of hPg to Sen is nonproductive when activated by the exogenous hPg activator SK2b, which is coexpressed with PAM, but activation of hPg in the Sen/hPg complex is stimulated when host tPA is used as the activator.

## Data Availability Statement

The raw data supporting the conclusions of this article will be made available by the authors, without undue reservation.

## Author Contributions

YAA, ST-F, BMR, ZL, and OA performed experiments and generated the data. LNP, SWL, VAF, and VAP provided critical input for the experiments and their interpretation and reviewed the manuscript. FC conceptualized the study and wrote the manuscript. All authors contributed to the article and approved the submitted version.

## Funding

These studies were supported by grant HL013423 to FC, VAP, and SWL.

## Conflict of Interest

LNP is employed by BioAnalysis, LLC.

The remaining authors declare that the research was conducted in the absence of any commercial or financial relationships that could be construed as a potential conflict of interest.

## Publisher’s Note

All claims expressed in this article are solely those of the authors and do not necessarily represent those of their affiliated organizations, or those of the publisher, the editors and the reviewers. Any product that may be evaluated in this article, or claim that may be made by its manufacturer, is not guaranteed or endorsed by the publisher.

## Footnotes

^1^ http://www.rasmb.org/sednterp/

^2^ http://www.rasmb.org/sednterp/

^3^ http://www.rasmb.org/sednterp/

^4^ https://spsrch.cit.nih.gov/

## Supplementary Material

Supplementary Figure 1 Scanning Electron Microscopy (SEM) analysis of the presence of Sen on isogenic AP53 cells. GAS-AP53 cells were grown to mid-log phase and incubated with rabbit-anti Sen or hPg followed by mouse-anti hPg to determine the relative abundance of Sen on the surface of isogenic AP53 strains. The samples were imaged using the Magellan 400 FESEM to assess the abundance of bound antibody in different isogenic strains. **(A)** WT-AP53 cells without antibody. **(B)** WT-AP53 cells with rabbit-anti Sen. **(C)** WT-AP53 cells with hPg followed by mouse-anti hPg. **(D)** AP53/∆*pam* cells with rabbit-anti Sen. **(E)** AP53/*pam[8A]* cells with rabbit anti-Sen. **(F)** AP53/*pam[8A]* cells with hPg followed by mouse-anti hPg.

Supplementary Figure 2 SEM analysis of the presence of Sen on isogenic SF370 cells. GAS-SF370 cells were grown to mid-log phase and incubated with rabbit-anti Sen to determine the relative abundance of Sen on the cell surface. The samples were imaged using the Magellan 400 FESEM for determination of the abundance of bound antibody. **(A)** WT-SF370 cells with rabbit-anti Sen. **(B)** SF370/∆*M1* cells with rabbit-anti Sen. **(C)** Bar representation of median fluorescence intensity (MFI) obtained from FCA of the binding of rabbit-anti Sen to SF370 and SF370/∆*M1* cells as a function of the growth phase of the cells. OD_600nm_ of 0.3, 0.55, and 1 are early log-phase, mid log-phase, and stationery-phase of growth, respectively.
