# Supplementary figures and images for: Relationships Between Plasminogen-Binding M-Protein and Surface Enolase for Human Plasminogen Acquisition and Activation in Streptococcus pyogenes
Source: Front Microbiol. 2022 May 24;13:905670. doi: 10.3389/fmicb.2022.905670 (PMC9173704; doi:10.3389/fmicb.2022.905670)

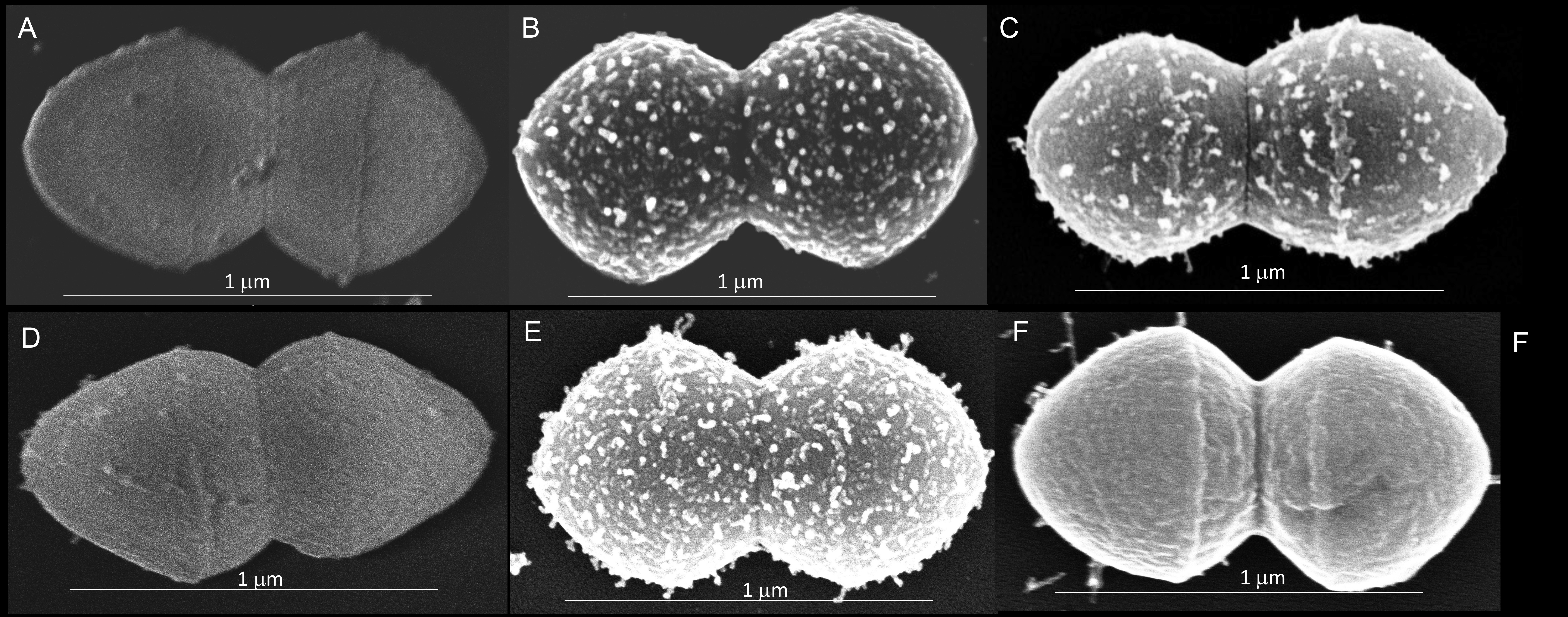

Supplement: Supplementary Figure 1 — Scanning Electron Microscopy (SEM) analysis of the presence of Sen on isogenic AP53 cells. GAS-AP53 cells were grown to mid-log phase and incubated with rabbit-anti Sen or hPg followed by mouse-anti hPg to determine the relative abundance of Sen on the surface of isogenic AP53 strains. The samples were imaged using the Magellan 400 FESEM to assess the abundance of bound antibody in different isogenic strains. (A) WT-AP53 cells without antibody. (B) WT-AP53 cells with rabbit-anti Sen. (C) WT-AP53 cells with hPg followed by mouse-anti hPg. (D) AP53/∆pam cells with rabbit-anti Sen. (E) AP53/pam[8A] cells with rabbit anti-Sen. (F) AP53/pam[8A] cells with hPg followed by mouse-anti hPg. [file Image_1.JPEG]

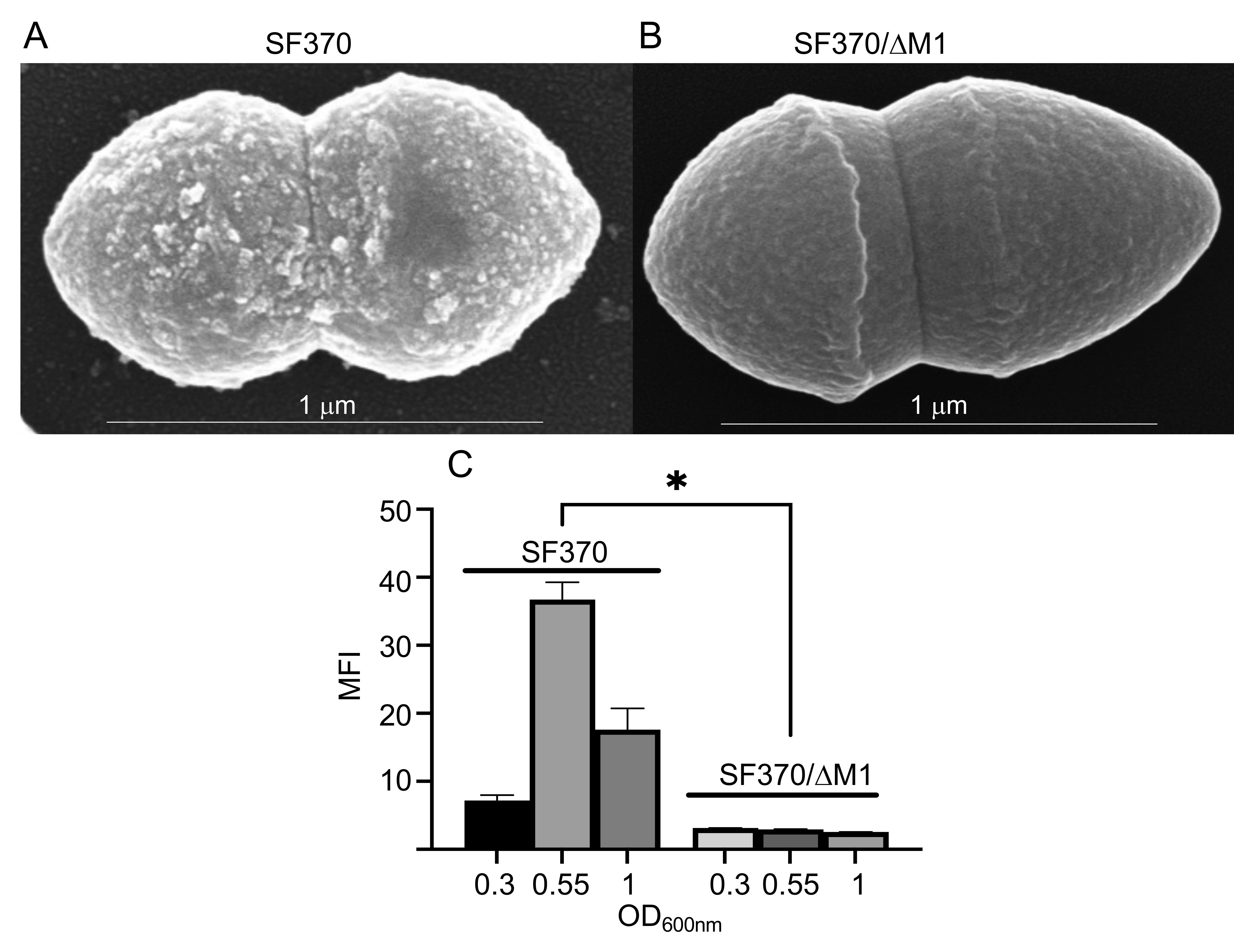

Supplement: Supplementary Figure 2 — SEM analysis of the presence of Sen on isogenic SF370 cells. GAS-SF370 cells were grown to mid-log phase and incubated with rabbit-anti Sen to determine the relative abundance of Sen on the cell surface. The samples were imaged using the Magellan 400 FESEM for determination of the abundance of bound antibody. (A) WT-SF370 cells with rabbit-anti Sen. (B) SF370/∆M1 cells with rabbit-anti Sen. (C) Bar representation of median fluorescence intensity (MFI) obtained from FCA of the binding of rabbit-anti Sen to SF370 and SF370/∆M1 cells as a function of the growth phase of the cells. OD600nm of 0.3, 0.55, and 1 are early log-phase, mid log-phase, and stationery-phase of growth, respectively. [file Image_2.JPEG]
